# Evaluation of the clinical application of information-motivation-behavior model care combined with labetalol pharmacotherapy for patients with hypertensive disorders of pregnancy: a randomized controlled study for improving pregnancy outcomes

**DOI:** 10.3389/fmed.2025.1627725

**Published:** 2025-10-21

**Authors:** Linling Sun, Jiamei Chen

**Affiliations:** Department of Obstetrics, Xuyi County People’s Hospital, Huai’an, Jiangsu, China

**Keywords:** hypertensive disorders of pregnancy, information-motivation-behavior model care, labetalol, self-efficacy, blood pressure control, negative emotions, pregnancy outcomes

## Abstract

**Background:**

The aim of this study was to assess the effect of Information-Motivation-Behavioral Model of Care (IMB) combined with labetalol medication on pregnancy outcomes in patients with hypertensive disorders of pregnancy (HDP).

**Methods:**

The study was a randomised, single-blind clinical trial with 130 patients with HDP admitted between February 2021 and February 2025, who were randomly assigned to the control and intervention groups. The control group received conventional labetalol medication, whereas the intervention group received a nursing intervention based on the IMB model. The IMB care consisted of the establishment of a dedicated nursing intervention team, informational interventions (e.g., health education), motivational interventions (e.g., personalised dietary life plan development), and behavioral interventions (e.g., guidance on self-monitoring of blood pressure).

**Results:**

IMB care combined with labetalol significantly increased patients’ self-efficacy, improved blood pressure control, and provided relief from negative emotions compared with labetalol alone. In addition, the intervention group had a longer gestational week of delivery (*p* = 0.04), less chance of vaginal delivery to caesarean section (*p* = 0.048), and a trend towards a decrease in the incidence of adverse pregnancy outcomes such as preterm labor and adverse effects such as nausea, albeit not statistically significant (*p* > 0.05).

**Conclusion:**

The IMB care model combined with labetalol medication had a positive impact on blood pressure control and pregnancy outcomes in patients with HDP, providing data support and theoretical basis for the application of this care model in HDP. However, the study also pointed out its limitations, such as single-centre design and limited sample size, and more comprehensive studies are needed to further validate these findings in the future.

## Introduction

Hypertensive disorders of pregnancy (HDP) is a condition specific to pregnancy characterised by changes in blood pressure, the presence of urinary proteins or involvement of vital organ systems. It occurs most often in mid-pregnancy and is characterised by a persistent increase in blood pressure after 20 weeks of gestation, which usually returns to normal within 12 weeks postpartum (1). Pregnant women with HDP may suffer from oedema, headache, visual impairment, proteinuria, and chest tightness, which in severe cases may lead to heart failure, cerebral haemorrhage, and other even life-threatening conditions (2, 3). Data statistics, as of 2019, there are 1,810 cases of HDP patients worldwide, affecting about 10% of pregnancies, of which 0.15% of deaths are accounted for (4, 5).

The global burden of HDP is substantial and may be increasing, in part due to trends in advanced maternal age and obesity (6). It is a leading cause of maternal mortality worldwide, particularly in low- and middle-income countries, and a significant contributor to severe maternal morbidity including stroke, organ failure, and disseminated intravascular coagulation (7). For the neonate, HDP is associated with elevated risks of intrauterine growth restriction, preterm birth, and stillbirth (8). Beyond the immediate perinatal period, HDP confers a lifelong elevated risk of cardiovascular disease for the mother (9), and offspring may face increased risks of metabolic and neurodevelopmental disorders (10). This significant morbidity translates into a considerable economic burden on healthcare systems, encompassing costs of prolonged hospitalization, intensive maternal and neonatal care, and long-term management of chronic conditions (11). Therefore, developing effective strategies to improve outcomes in HDP is not only a clinical imperative but also a significant public health priority.

HDP patients are prone to myocardial ischaemia and myocardial injury due to hypertension and inadequate blood circulation. In patients with HDP, oral antihypertensive drugs are often used clinically to control blood pressure and prevent adverse pregnancy outcomes (12). Labetalol is the clinically preferred drug for lowering blood pressure in patients with HDP, and its antihypertensive and cardioprotective effects have been widely recognised as a non-selective *β*-blocker (13). Studies have shown that labetalol can reduce blood pressure and cardiac load by inhibiting the activity of the sympathetic nervous system and reducing cardiac contractility and heart rate (14). In terms of anti-inflammatory and antioxidant effects, labetalol can reduce the inflammatory response and cellular damage, and improve the symptoms and conditions of HDP patients (15). However, the effect of blood pressure control is closely related to the patients’ own life behaviors and habits, and most of the patients often suffer from unsatisfactory blood pressure control due to the lack of correct knowledge of the disease and their own bad habits. Therefore, in addition to clinical symptomatic treatment, health education should be strengthened to correct patients’ bad habits, so that they can follow the doctor’s instructions to use medication, effectively control blood pressure, and prevent poor prognosis (16).

Information-Motivation-Behavioral Skill (IMB Model) is a theory of behavioral change proposed by the American researcher Fisher and continuously improved on the basis of other behavioral theories, which has been applied and developed in many fields and has strong applicability (17). Hua et al. found that an IMB model intervention for people with gestational weight gain was effective in managing weight and reducing adverse maternal and infant outcomes (18). Vamos et al. conducted an IMB model-based intervention for prenatal oral care for pregnant women, and showed that the model improved their perceptions and shaped their oral health behaviors (19). Nelson et al. found that an IMB intervention for patients with type 2 diabetes increased medication adherence and improved hemoglobin (20). However, its application in HDP care remains unexplored. Therefore, this study is the first randomized controlled trial to investigate the synergistic effect of a structured IMB model-based care integrated with standard labetalol pharmacotherapy on improving pregnancy outcomes in patients with HDP. We hypothesized that this combined approach would enhance patient self-efficacy and adherence, leading to superior blood pressure control and ultimately, better maternal and neonatal outcomes.

We aimed to investigate whether the IMB model of care combined with labetalol medication has an improved effect on pregnancy outcomes in patients with HDP, focusing on exploring the gainful effect of IMB care on top of labetalol medication.

## Methods

### Study design

The present study was a randomised, single-blind study of 130 patients with HDP in a randomised group with the aim of evaluating the effect of the IMB model of care in combination with labetalol drug therapy in improving pregnancy outcomes in patients with HDP. All study subjects and their families signed an informed consent form prior to enrolment. This trial was registered at the Chinese Clinical Trial Registry (www.chictr.org.cn; registration number: ChiCTR2100051022).

### Participants

One hundred and thirty HDP patients admitted to our hospital from February 2021–February 2025 were selected as the study subjects. Inclusion criteria: (1) meeting the diagnostic criteria of HDP and the first appearance of hypertension after 20 weeks of pregnancy; (2) informed consent of the mothers and their families to this study. Exclusion criteria: (1) with cardiac, hepatic and renal dysfunction and primary cardiovascular and cerebrovascular diseases; (2) combined with endocrine diseases such as hyperthyroidism and diabetes mellitus; (3) allergy to labetalol; (4) malignant tumor; (5) suffering from severe mental disorders.

We randomised the order of admission of the study subjects by serial number with reference to the random number table method, and the first 65 patients of the generated serial number of the arrangement were taken as the control group, and the 66–130 patients were taken as the intervention group.

### Sample size calculation

An *a priori* sample size calculation was performed using G*Power software (version 3.1). Based on a previous pilot study and published literature (21), we anticipated that the primary outcome of self-efficacy (as measured by the PIH scale) would show a mean difference of approximately 4 points between groups, with a standard deviation of 5.5. Setting a two-sided alpha (*α*) level of 0.05 and a desired power (1-*β*) of 0.90, the calculation yielded a minimum required sample size of 66 patients per group. To account for a potential dropout rate of up to 5%, we aimed to recruit a total of 140 patients. The final analysis included 65 patients per group (130 total), which meets the calculated requirement for the primary outcome.

### Study setting and standard care

This study was conducted within the standard framework of obstetric care for HDP at our institution. It is critical to clarify that participants were not required to be hospitalized for the entire duration of the study. The IMB nursing interventions and labetalol pharmacotherapy were primarily administered and followed up in an outpatient clinic setting. Hospital admission was reserved for and triggered solely by standard obstetric indications reflecting worsening maternal or fetal condition. These indications included: (1) severe hypertension (systolic blood pressure ≥160 mmHg or diastolic blood pressure ≥110 mmHg) despite antihypertensive therapy; (2) clinical signs of impending severe preeclampsia (e.g., severe headache, visual disturbances, epigastric pain); (3) significant abnormal laboratory values (e.g., thrombocytopenia, elevated liver enzymes); (4) suspected fetal growth restriction or abnormal fetal heart rate patterns; or (5) reaching term gestation for planned delivery.

All patients enrolled in the trial, irrespective of their group allocation (control or intervention), received the same standard of background care according to prevailing institutional guidelines. This baseline management included: regular blood pressure monitoring, serial proteinuria assessment (dipstick or 24-h collection), fetal well-being surveillance (via cardiotocography and ultrasonography for biophysical profile and Doppler studies as indicated), and dietary advice emphasizing a low-sodium diet. Crucially, if a patient developed signs of severe preeclampsia or eclampsia, she immediately received magnesium sulfate infusion for seizure prophylaxis, following standard protocols. The experimental comparison was therefore between ‘Standard Care + Labetalol’ (control group) and ‘Standard Care + Labetalol + IMB Model Nursing’ (intervention group).

### Intervention

Both groups of patients were given routine treatment such as sedation, real-time monitoring of foetal heart and review of proteinuria after admission. The control group was given labetalol (Jiangsu Dixie Nuo Pharmaceutical Co., Ltd., 50 mg/tablet, State Pharmaceutical License H32026120), which was taken orally after meals, 2 tablets/times/day, and treated continuously for 1 month.

The intervention group was given IMB nursing on this basis, and the nursing measures were as follows:

(1) Establishment of the IMB model nursing intervention team: the intervention team consisted of two obstetricians, one psychological counsellor, one nutritionist, two nurses and two midwives. The midwives and nurses were responsible for the development and implementation of the IMB nursing intervention process, and the obstetricians adjusted the nursing intervention strategy according to the clinical reality to ensure the feasibility and rationality of the strategy. (2) Information intervention: After the patients were admitted to the hospital, the nurses established good communication with the patients, adopted a gentle and patient attitude towards the patients, and answered the questions raised by the patients in a timely manner. Interviews were organised once a week to understand the patients knowledge of the disease and their daily lifestyle, and to analyse the high-risk factors according to the patients’ past history and baseline blood pressure. Through questions and discussions, we systematically learnt about hypertension in pregnancy and nursing interventions, developed personalised education, and collected the knowledge and existing problems of all patients at different stages during the health education period. Online, we established a WeChat group for patients and nurses, regularly releasing audio-visual educational resources on the topic of hypertension in pregnancy and completing the Q&A work for patients. (3) Motivational intervention: Dietitians and psychological counsellors formulate personalized dietary and lifestyle plans for each patient in the group, and provide targeted psychological guidance based on the complaints of patients who have difficulties in implementing self-management. Patients who have good blood pressure control are invited to share their experiences with the group, so as to build an atmosphere of mutual support and encouragement among patients. For patients with good blood pressure management and significant lifestyle changes, certain incentives will be given to promote the development of healthy living habits. (4) Behavioral intervention: according to the patients’ awareness of the disease, their own concepts and bad behaviors, health education programmes are formulated to encourage them to change their concepts and develop their own healthy behaviors from their subjective consciousness. Psychological counselling was provided to patients to explain the influence of their emotions on blood pressure fluctuations and to maintain emotional stability. Instruct patients to learn how to monitor their own blood pressure and take medication as prescribed by the doctor, in order to better control their blood pressure and reduce the risk of complications. Assist patients to formulate personalised care plans according to their recent status and instruct them to consume more food rich in protein and vitamins, and to eat a low-fat and low-salt diet. Safe exercise options such as walking and yoga for pregnant women were recommended. Oxygen therapy was not routinely administered. It was provided only if clinical signs of fetal distress (e.g., non-reassuring fetal heart rate tracing) were observed. In such cases, supplemental oxygen was delivered via a face mask at a flow rate of 5–8 L/min for 20–30 min, following standard clinical practice to improve fetal oxygenation. This intervention is up to normal delivery.

### Evaluation of indicators

Timing of assessments: Outcome measures were collected at the following time points: (1) Baseline: upon study enrollment (after admission); (2) Post-intervention: after completing the 1-month intervention protocol or immediately prior to delivery if it occurred earlier; (3) At delivery: recorded at the time of birth; (4) Postpartum: assessed prior to hospital discharge after delivery.

Primary outcomes: The self-efficacy of the two groups was analysed using the Partners in Health (PIH) Scale (21), which consists of three dimensions: knowledge, coping and adherence, with a total of 12 entries. Each entry was scored out of 8 points, with a total score of 56 points for knowledge, 24 points for coping and 16 points for adherence, which were positively correlated with self-management ability. This was assessed at Baseline and Post-intervention time points.

The pregnancy indices were measured at Baseline and Post-intervention time points and analysed and compared between the two groups: SBP, DBP, urinary protein, end-systolic maximal blood flow rate/end-diastolic minimal blood flow rate (S/D) of the umbilical artery of the placenta, pulsatility index (PI) and resistance index (RI).

Deliveries were counted separately at the time of birth, including gestational week of delivery, mode of delivery and adverse pregnancy outcomes. Adverse pregnancy outcomes included preterm labor, postpartum haemorrhage, placental abruption, neonatal asphyxia and fetal distress.

The incidence of adverse effects, including nausea, vomiting, eye discomfort, lower limb oedema and palpitations, were monitored throughout the entire treatment period and counted separately at the Post-intervention assessment.

The negative emotions of the patients were assessed at Baseline and Post-intervention time points using the Self-Assessment Scale for Anxiety (SAS) and the Self-Depression Scale (SDS).

Secondary outcomes: Satisfaction with nursing care was assessed at the Postpartum time point using a self-made satisfaction questionnaire with a score of 100, with more than 85 indicating great satisfaction, 60–85 indicating basic satisfaction, and less than 60 indicating dissatisfaction.

### Statistical analysis

**Figure 1:**
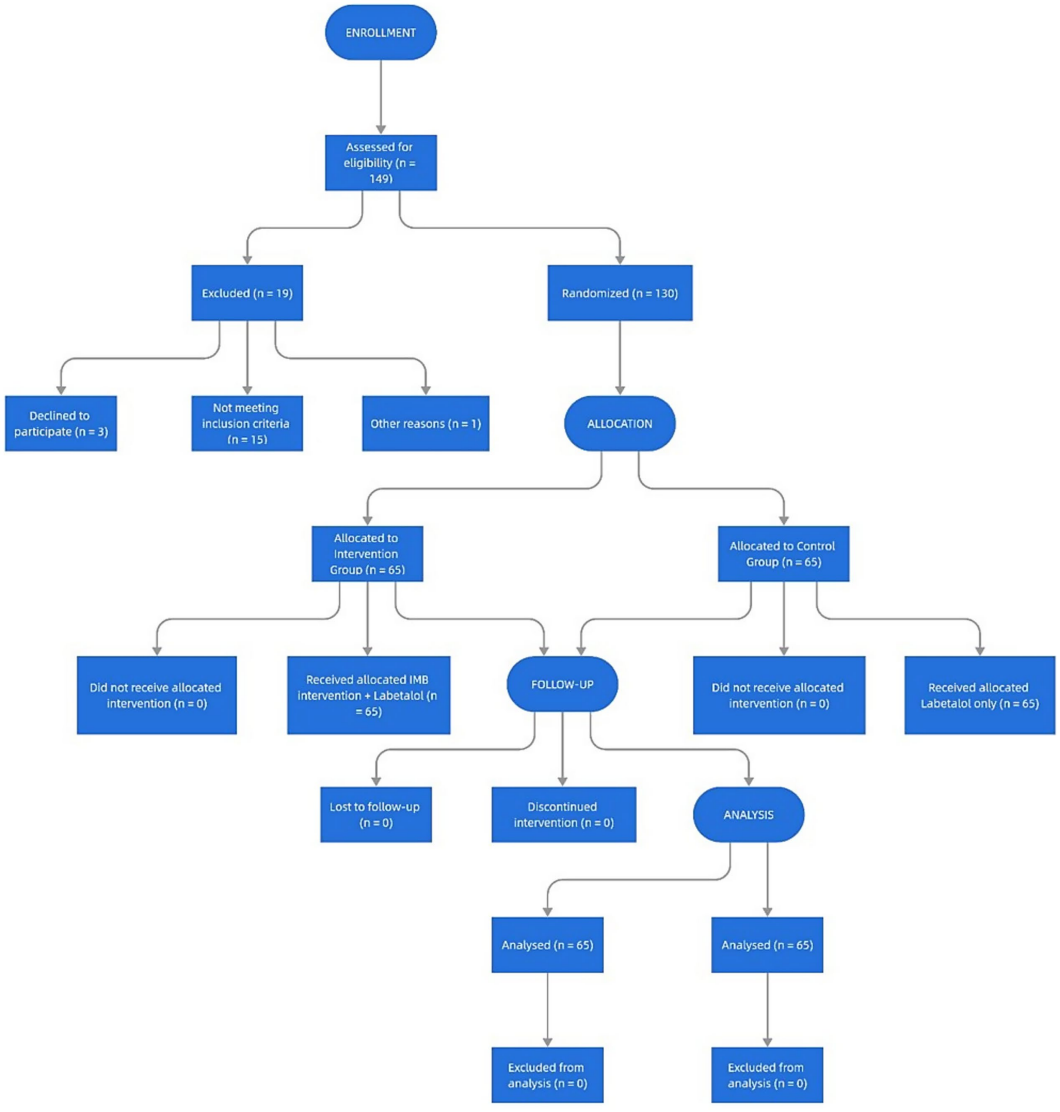
Flow diagram of the progress through the phases of the randomized trial.

In order to test whether there was any difference in the variables between the groups, quantitative analyses were carried out using the χ^2^ test in SPSS 21.0 according to the test conditions. All normally distributed continuous data were analysed using student’s t test while non-parametric data were analysed using Mann–Whitney U test. Count data and measured data are expressed as *n* (%) and mean ± SD, respectively, and all tests were two-sided. *p* < 0.05 was considered a statistically significant difference (see Figure 1).

## Results

The 130 mothers included in the study had a mean age of 28.54 and mean gestational week of 31.25 at the time of admission, and more than 75% of the mothers were pregnant for the first time. They were divided into Control and Intervention groups as per the requirement, there was no statistical difference in blood pressure, urine protein level before intervention between the two groups and they were comparable (*p* > 0.05, Table 1).

**Table 1:** Baseline characteristics of maternity (mean ± SD) or *n* (%).

Parameter		Control	Intervention	X^2^ (t)	*P*
Number		65	65	-	-
Age (years)		27.69 ± 4.98	29.15 ± 4.28	1.793	0.075
Weeks of gestation on admission (weeks)		31.18 ± 2.2	31.42 ± 1.96	0.657	0.513
BMI (kg/m^2^)		22.21 ± 2.45	21.97 ± 2.36	0.569	0.571
Maternal history, *n* (%)	First pregnancy	53 (81.54)	49 (75.38)	0.728	0.393
	Multiple pregnancies	12 (18.46)	16 (24.62)		
Place of residence, *n* (%)	Town	46 (70.77)	52 (80.00)	1.492	0.222
	Village	19 (29.23)	13 (20.00)		
Educational attainment	High school and below	17 (26.15)	13 (20.00)	0.938	0.626
	College or Bachelor’s Degree	38 (58.46)	39 (60.00)		
	Postgraduate and above	10 (15.39)	13 (40.00)		
SBP (mmHg)		162.24 ± 16.57	160.95 ± 18.87	0.414	0.679
DBP (mmHg)		105.22 ± 12.31	104.19 ± 11.68	0.489	0.625
Urinary protein (mg/24 h)		752.55 ± 20.69	753.28 ± 19.65	0.206	0.837

**Figure 2:**
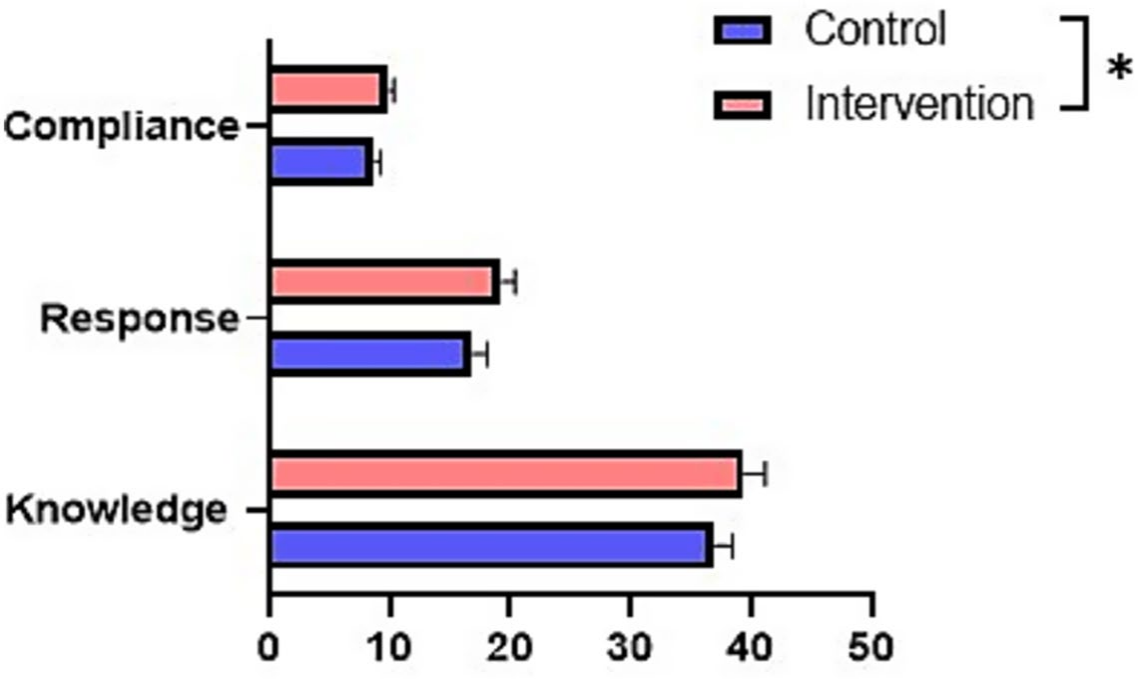
Comparison of post-intervention self-efficacy between the two groups. *P < 0.05.

In self-efficacy, patients in the intervention group scored higher than the control group in knowledge (39.42 ± 1.75 vs. 36.97 ± 1.52, *p* < 0.001), coping (19.24 ± 1.16 vs. 16.83 ± 1.28, *p* < 0.001), and adherence (9.88 ± 0.47 vs. 8.75 ± 0.53, *p* < 0.001), which indicated that the IMB care was effective in improving self-efficacy in patients with gestational hypertension (Figure 2).

**Figure 3:**
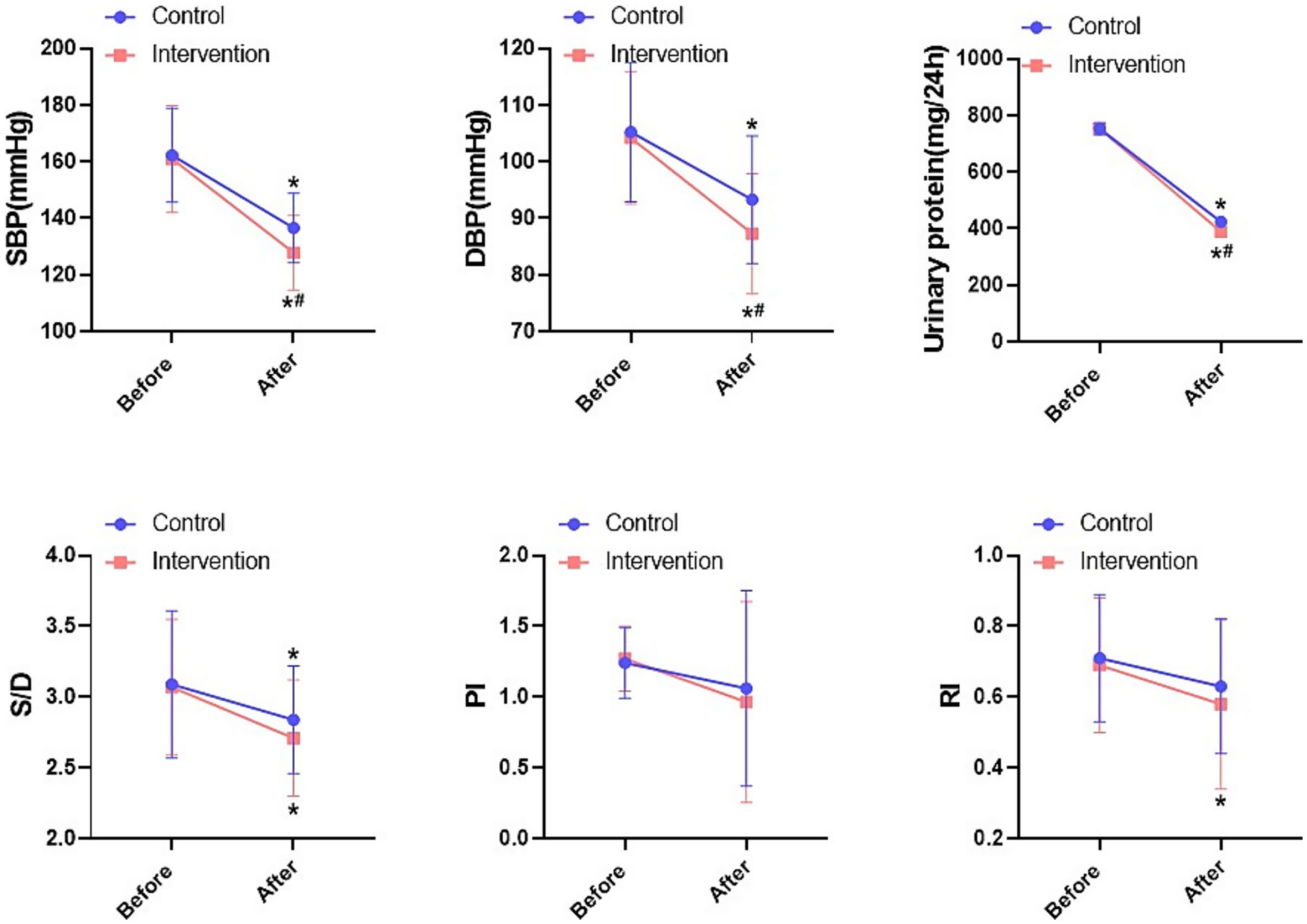
Comparison of blood pressure indexes and haemodynamic indexes between the two groups. *P < 0.05, * compared with before; #*P* < 0.05, # compared with control.

Pregnancy indicators showed that SBP, DBP and urinary protein levels were significantly reduced in both groups compared with the pre-intervention period, with a more pronounced reduction in the intervention group (*p* < 0.05). In terms of haemodynamic indicators, there was a significant reduction in S/D after the intervention, but there was no statistical difference between the two groups (*p* > 0.05, Figure 3). Overall, the IMB care model combined with labetalol was more effective in controlling patients’ blood pressure.

In the delivery situation and adverse effects, the gestational week of delivery in the intervention group was around 37.18 weeks, compared with 36.59 weeks in the control group, which was a significant increase in gestational week (*p* = 0.004). There was a significant decrease in the number of failed vaginal deliveries converted to caesarean section in the intervention group compared to the control group (*p* = 0.048, Table 2). In addition, the overall incidence of adverse pregnancy outcomes such as preterm labor, placental abruption etc. in the intervention group showed a decreasing trend compared to the control group but was statistically insignificant (12.31% vs. 21.54%, *p* = 0.161, Table 3). This suggests that the IMB care model combined with labetalol has improved the pregnancy outcome of patients with gestational hypertension to some extent. Among the adverse effects, the overall incidence of adverse effects such as nausea, headache etc. in the intervention group showed a decreasing trend as compared to the control group but it was statistically insignificant (9.23% vs. 16.92%, *p* = 0.193, Table 4). This suggests that the IMB care model combined with labetalol is a safer treatment strategy and there is a potential improvement in adverse effects in patients with gestational hypertension.

**Table 2:** Statistics on the delivery of patients in the two groups.

Groups	Gestational age at delivery (weeks, Mean ± SD)	Mode of delivery [*n* (%)]		
		Vaginal delivery	Caesarean section	Vaginal delivery to caesarean section
Control (*n* = 65)	36.59 ± 1.06	37 (56.92)	20 (30.77)	8 (12.31)
Intervention (*n* = 65)	37.18 ± 1.24	44 (67.69)	19 (29.23)	2 (3.08)
X^2^ (t)	2.916	1.605	0.037	3.900
*P*	0.004	0.205	0.848	0.048

*P* < 0.05 compared to the control group.

**Table 3:** Adverse pregnancy outcomes in the two groups [*n* (%)].

Outcomes	Control (*n* = 65)	Intervention (*n* = 65)	*P*
Preterm labor	7 (10.77)	5 (7.69)	0.547
Postpartum hemorrhage	5 (7.69)	2 (3.08)	0.443
Placental abruption	4 (6.15)	2 (3.08)	0.680
Neonatal asphyxia	3 (4.62)	1 (1.54)	0.617
Fetal distress	5 (7.69)	3 (4.62)	0.715
Total incidence	14 (21.54)	8 (12.31)	0.161

Differences between groups were compared using the Chi-square test or Fisher’s exact test as appropriate.

**Table 4:** Adverse reactions in the two groups [*n* (%)].

Adverse reactions	Control (*n* = 65)	Intervention (*n* = 65)	*P*
Nausea	3 (4.62)	2 (3.08)	1.000
Vomiting	1 (1.54)	0 (0.00)	1.000
Eye discomfort	1 (1.54)	2 (3.08)	1.000
Lower limb edema	2 (3.08)	1 (1.54)	1.000
Palpitations	1 (1.54)	0 (0.00)	1.000
Headache	2 (3.08)	1 (1.54)	1.000
Total incidence	11 (16.92)	6 (9.23)	0.193

Differences between groups were compared using the Chi-square test or Fisher’s exact test as appropriate. *p*-values were calculated for each individual adverse reaction and for the total incidence.

To account for potential confounding, multivariate logistic regression analyses were conducted. After adjusting for maternal age, baseline systolic blood pressure, and primiparity, the intervention group maintained a significantly lower risk of conversion from vaginal delivery to caesarean section (Adjusted Odds Ratio [aOR] = 0.23, 95% Confidence Interval [CI]: 0.05–0.99, *p* = 0.049). For the composite outcome of adverse pregnancy outcomes, the intervention group showed a strong trend towards a reduced risk, although it did not reach statistical significance in the adjusted model (aOR = 0.52, 95% CI: 0.20–1.35, *p* = 0.178). The results of the regression analyses are presented in Table 5.

**Table 5:** Multivariate logistic regression analysis of primary outcomes.

Outcome variables	Adjusted odds ratio (aOR)	95% confidence interval	*P*
Conversion to caesarean section	0.23	0.05–0.99	0.049
Composite adverse pregnancy outcomes	0.52	0.20–1.35	0.178

Models were adjusted for maternal age, baseline systolic blood pressure, and primiparity status. aOR, adjusted odds ratio.

**Figure 4:**
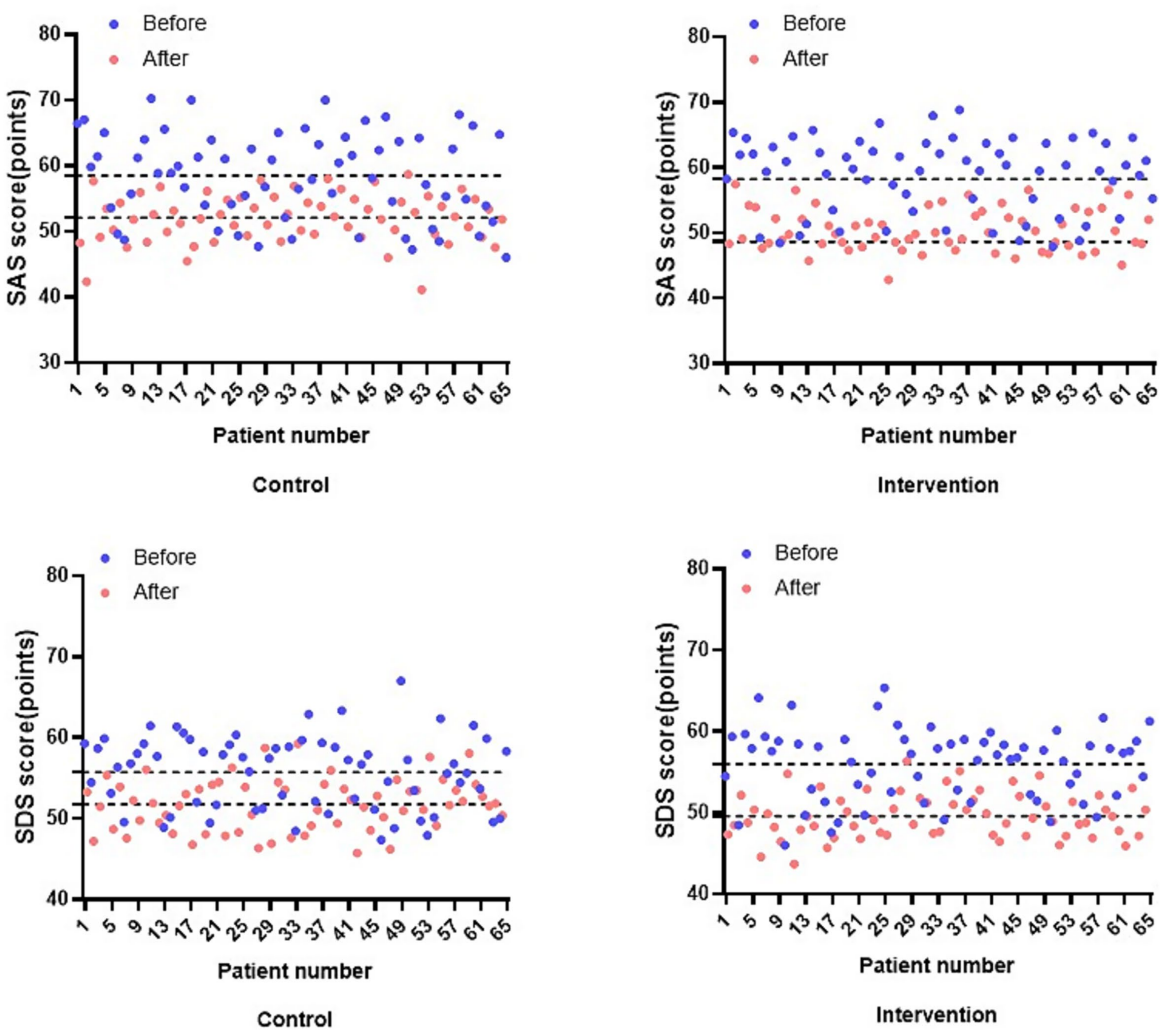
Comparison of SAS and SDS scores before and after the intervention in both groups.

In the assessment of negative mood, SAS in the control group decreased from (58.48 ± 6.66) to (52.06 ± 3.72) and SDS from (55.71 ± 4.54) to (51.75 ± 3.23) before intervention, which was statistically significant (*p* < 0.001). SAS in the intervention group decreased from (58.68 ± 5.73) to (50.44 ± 3.28) and SDS from (55.97 ± 4.38) to (49.56 ± 2.72) in the pre-intervention group, which was statistically significant (*p* < 0.001). There was also a significant decrease in SAS (*p* = 0.010) and SDS (*p* = 0.002) in the intervention group as compared to the control group after the intervention (Figure 4). Regarding a secondary exploratory endpoint, patients in the intervention group reported higher overall satisfaction with the care they received compared to the control group (see Supplementary Table S1 for detailed results).

## Discussion

In patients with HDP, IMB care combined with labetalol significantly improved clinical blood pressure control and self-efficacy compared with labetalol therapy alone, with varying degrees of improvement present for negative mood and pregnancy outcomes. This provides data support and additional possibilities for clinical translation of IMB care combined with labetalol in HDP care.

Here, IMB care combined with labetalol significantly increased self-efficacy in HDP patients (*p* < 0.001). Self-efficacy was originally proposed by scholar Bandura as a dynamic cognitive process that refers to an individual’s level of confidence in his or her ability to achieve a particular event (22), whereas self-efficacy during pregnancy refers to a pregnant woman’s confidence and beliefs in her ability to lower her blood pressure and improve the outcome of her pregnancy. This is due to the fact that IMB care is effective in meeting the patient’s knowledge needs, changing the patient’s perception, and focusing on improved outcome expectations, which leads to improved treatment adherence. Adherence is an important influence on improving the prognosis of patients with HDP, and in recent years, the development of several emerging technologies has emerged, Spencer et al. improved the efficiency of timely medication administration and adherence to HDP guidelines by constructing automated electronic health record alerts (23). Rajkumar et al.’s improved adherence to blood pressure follow up in patients with HDP and improved labor outcomes through antenatal remote blood pressure monitoring (24). However, the availability and importance of health education is a fundamental measure to promote good treatment adherence, and the results of a qualitative study of hypertensive patients revealed that lack of hypertension awareness reduces the importance of hypertension management and thus medication adherence (25). Several studies have demonstrated that health education is an important measure to enhance self-efficacy (26, 27).

Here, IMB care combined with labetalol significantly improved physiological indices of HDP including SBP, DBP, urinary protein and M/D (*p* < 0.05). It is well known that gestational hypertension leads to placental vasospasm and increased resistance, affecting placental umbilical blood perfusion. Labetalol, a salicylamide derivative, reduces blood pressure and improves placental blood circulation. Therefore, the two groups of patients will have different degrees of improvement in blood pressure, urinary protein level and haemodynamics after the intervention (*p* < 0.05). Pirponen et al. found that the uterine artery S/D ratio decreases in HDP patients with reduced blood pressure, which also suggests the potential improvement of fetal haemodynamics by the lowering of maternal blood pressure (28). This is in line with our findings, however, there was no significant effect of labetalol treatment on PI and RI indices (*p* > 0.05). Due to individual differences, the effect of labetalol on fetal haemodynamics is not conclusive at present, Harper et al. found that there would be a tendency for some foetuses to show an increase in PI (29). Mowafy et al. observed the cerebral haemodynamics of patients with pre-eclampsia treated with labetalol, and it was found that there was a significant decrease in PI as soon as one hour after the administration of the drug (30). Thus, the effect of labetalol on the haemodynamics of different individuals is variable. IMB care combined with labetalol showed better improvement in blood pressure and urinary protein compared to treatment alone, which may be more related to the improvement in patient self-efficacy.

Here, the gestational week of delivery in the intervention group was longer compared with that in the control group, which may be due to the fact that IMB care combined with labetalol can significantly improve the placental umbilical blood flow indexes, and the blood perfusion of the placenta is good, which provides the fetus with sufficient nutrients and oxygen, which is conducive to the growth and development of the fetus in the uterus and reduces the risk of preterm delivery caused by placental dysfunction, thus prolonging the gestational week of delivery. A key finding of our study was the significantly reduced rate of conversion from planned vaginal delivery to caesarean section in the intervention group compared to the control group (3.08% vs. 12.31%, *p* = 0.048). This finding was further confirmed by multivariate logistic regression analysis (aOR = 0.23, *p* = 0.049), underscoring the independent benefit of the IMB intervention. We hypothesize that this reduction may be attributed to several mechanisms facilitated by the IMB model: (i) Improved maternal physiological status: Better blood pressure control and potentially improved utero-placental function may have reduced the incidence of intrapartum fetal distress, which is a primary indication for emergency caesarean in HDP. (ii) Enhanced maternal autonomy and preparedness: The informational and motivational components of the intervention likely reduced anxiety and increased the patient’s sense of control and commitment to a vaginal birth, potentially leading to more perseverance during the first stage of labor. (iii) Optimized clinical decision-making: With patients being more engaged and informed, communication with healthcare providers may have been more effective, leading to more nuanced and less rushed decisions regarding delivery mode. To contextualize this finding, the overall caesarean section rate in our study (approximately 30% in both groups) remains lower than the reported national average in China, which has been among the highest globally, exceeding 40% in many regions (31). However, it still far exceeds the WHO recommended rate of 10–15% (32). Therefore, while the IMB intervention resulted in a clinically meaningful relative reduction, there is a continued imperative for broader strategies to address the high absolute baseline rate of caesarean delivery in our setting. Our study suggests that structured patient education and support programs could be one such valuable strategy. The success of the IMB model in this predominantly outpatient setting demonstrates its potential as a feasible and effective strategy to bridge the gap between intermittent clinical visits and continuous self-management for HDP patients.

Molvi et al. used labetalol to treat patients with PDH, and the cesarean section rate of such treated patients was significantly reduced compared with that of the patients who received standard care, confirming the critical role of blood pressure control on pregnancy outcome (33). Among the adverse effects, several studies have confirmed the safety of clinical use of labetalol, which is one of the reasons why labetalol can be used as a first-line drug (34). In our study, IMB care in combination with labetalol caused fewer cases of adverse reactions compared to labetalol alone, but statistically insignificant (*p* = 0.169), which confirms the high safety of combined therapeutic care.

Studies have shown that the subjective well-being of pregnant women is positively correlated with social support, and the less negative emotions they feel, the less pregnancy stress and postpartum depression they experience (35, 36). The IMB model of care can help patients with HDP to establish a correct cognition, stabilise their emotions, and provide them with an objective and practical way of self-management. In addition, through the care and support of healthcare professionals, family members, and peers, it gives support in social interactions and helps to detoxify negative emotions and promote psychological well-being. This is the reason for better improvement of anxiety and depression in patients who received IMB care combined with labetalol treatment in this study (*p* < 0.05). IMB care has also been shown to be effective in improving negative emotions in patients with cesarean section (37), vestibular dysfunction (38), and cervical cancer (39), thereby reducing the condition and promoting recovery.

The study was a single-centre study and the number of studies was limited, which may cause some bias to the results. Secondly, HDP patients objectively have the complexity of physiopathological factors, and the basis of physiological indicators may vary greatly from patient to patient. With the progression of pregnancy, patients’ physiological indicators may undergo various changes. In this study, the main focus was on the changes in blood pressure and haemodynamic levels in HDP patients before and after receiving the interventions, however, the assessment of other physiological indicators such as coagulation, liver and kidney functions was lacking. Therefore, future studies should more comprehensively assess the impact of multiple physiological indicators including therapeutic agents and medication use. Furthermore, although we employed multivariate regression to adjust for key confounders, our sample size may have limited the power to include more variables in the model or to develop a full predictive nomogram. Building upon these findings, future studies with larger sample sizes are warranted to both confirm our results and to develop predictive models that can identify which subsets of HDP patients are most likely to benefit from this integrated care approach. Moreover, the assessment of nursing satisfaction, while indicating a positive response, was measured using a non-validated, self-made questionnaire. Although this tool was practical for our clinical context, the lack of formal validation limits the interpretation and generalizability of this particular finding. We recommend that future studies investigating similar interventions employ standardized, psychometrically validated instruments (e.g., the Patient Satisfaction Questionnaire Short Form [PSQ-18] or a specific maternity care satisfaction tool) to more robustly and reliably capture this outcome. Finally, negative emotions in patients with HDP are long-lasting, and due to factor limitations, long-term follow-up of patients was not conducted.

## Conclusion

IMB care combined with labetalol had the most significant improvement in blood pressure and pregnancy outcomes in HDP patients. Therefore, we recommend that patients with HDP receive professional nursing care along with antihypertensive medications to improve cognition and adherence, thereby improving their condition and pregnancy outcomes. The results of this study provide data support and theoretical guidance for the clinical translation of IMB care combined with labetalol therapy.

## Data Availability

The original contributions presented in the study are included in the article/Supplementary material, further inquiries can be directed to the corresponding author.
