# Supplementary material for: Evaluation of the clinical application of information-motivation-behavior model care combined with labetalol pharmacotherapy for patients with hypertensive disorders of pregnancy: a randomized controlled study for improving pregnancy outcomes
Source: Front Med (Lausanne). 2025 Oct 21;12:1627725. doi: 10.3389/fmed.2025.1627725 (PMC12582955; doi:10.3389/fmed.2025.1627725)
Supplement: Supplementary file 1 [file Table_1.docx]

**Supplementary Table S1 Nursing satisfaction scores**

| **Groups** | **Nursing satisfaction score (mean ± SD)** | **P** |
| --- | --- | --- |
| Control (n=65) | 77.87 ± 7.50 | 0.038 |
| Intervention (n=65) | 80.76 ± 8.19 |  |

Note: Satisfaction was assessed using an institutional self-made questionnaire (score range: 0-100). A higher score indicates greater satisfaction. The difference between groups was assessed using Student's t-test.
